# Supplementary material for: Can Clinical Assessment of Postural Control Explain Locomotive Body Function, Mobility, Self-Care and Participation in Children with Cerebral Palsy?
Source: Healthcare (Basel). 2024 Jan 1;12(1):98. doi: 10.3390/healthcare12010098 (PMC10779062; doi:10.3390/healthcare12010098)
Supplement: Supplementary file 1 [file healthcare-12-00098-s001.zip › healthcare-2779560-supplementary.pdf]

Supplementary Table S1. Locomotor Stages (LS)	
**Pathologic Locomotor Stages**	**Ontogenetic Locomotor Stages**
**STAGE 0**: **ORIENTATION**. The child´s motor performance is lower than in stage 1. In the supine position the child cannot grasp or turn its head or body in order to establish a contact.	Holokinetic movement
**STAGE 1**: **REACHING**. The child can turn his head and body towards a stimuli, and at least one hand tries to reach out with palmar flexion and cubital tilt. Grasping is restricted to the finger tips.	Reach out with hands towards toy. 4 month
**STAGE 2**: **GRASPING**. In prone position	Prone on forearms, weight on one side and able to grasp with the other hand. 4,5 months
**STAGE 3**: **CREEPING**. Child is able to move forward propping on one or both forearms. Legs may move in association, but are not able to push to create any locomotion.	Creeping or commando crawling. 7 months
**STAGE 4**: **HOMOLOGOUS CRAWLING**. Bunny hopping. Legs move forward at the same time, while arms can do it in a homologous or alternating way.	No analogy
**STAGE 5**: **RECIPROCAL CRAWLING**. Crawling on knees and open hands with a cyclic and sinusoidal shift of the centre of gravity around the midline. All extremities step forward alternating in a crossed pattern. This pattern is used by the child independently, without being told and when it moves quickly.	Reciprocal crawling. 9 months.
**STAGE 6**: **CRUISING**. The child can pull to a stand and walk sideways independently by holding onto furniture with his hands. Pull to stand may already appears at stage 3, but the child cannot step sideways.	Sideways walking/ cruising on the furniture. 12 -13 months
**STAGE 7**: **INDEPENDENT WALKING**. The child can walk forward and stop without holding on. Walking is the everyday preferred locomotion.	Walking, stop and turn without holding on. 15 months
**STAGE 8**: **UNILATERAL STANCE ON ONE SIDE**. The child can stand during 3 seconds or more only on one preferred side, left or right foot. The examiner must not demonstrate this test, but give instructions: “lift one knee up towards your belly”	Single Leg stance on right or left foot. 3 years
**STAGE 9**: **RECIPROCAL UNILATERAL STANCE**. The child is able to stand reciprocally during 3 seconds or more on both sides, right and left. The examiner gives the same instruction as in stage 8.	Single Leg stance on right and left foot. 4 years
